# Editorial: Insights in developmental endocrinology: 2023

**DOI:** 10.3389/fendo.2024.1453023

**Published:** 2024-08-01

**Authors:** Lawrence M. Nelson, Mayank Choubey, Hiroyasu Kamei, Christine Rampon

**Affiliations:** ^1^ Digital Women’s Health, Initiative Mary Elizabeth Conover Foundation, Tysons, VA, United States; ^2^ Department of Foundations of Medicine, New York University (NYU) Grossman Long Island School of Medicine, Mineola, NY, United States; ^3^ Institute of Science and Engineering, Kanazawa University, Kanazawa, Ishikawa, Japan; ^4^ Laboratoire des Biomolécules, École Normale Supérieure, PSL University, Centre National de la Recherche Scientifique, Sorbonne Université, Paris, France; ^5^ Faculty of Sciences, Université Paris-Cité, Paris, France

**Keywords:** developmental endocrinology, wisdom of the body, homeostasis, integrative biology, maternal fetal interface, women’s hormonal health, hormonal literacy, science advocacy

## Integrative developmental endocrinology

*The wisdom of the body perspective* transcends our current human understanding and is a call for more innovative biomedical research. Developmental endocrinology is integrative biology, involving the concept of homeostasis, and the elegant underpinnings of life itself (1). Developmental endocrinology involves the intricate relationship between maternal nutrition and offspring health and this has been the subject of extensive research and scientific inquiry (2). Miles et al., in a mouse model, investigate the effects of maternal caloric restriction in mid-gestation and lactation on neonatal development and adult metabolic function in response to a high-fat diet. Studies investigating the impact of maternal caloric restriction during specific stages of gestation and lactation shed light on the long-term implications for offspring health and adult metabolic function.

**Figure 1:**
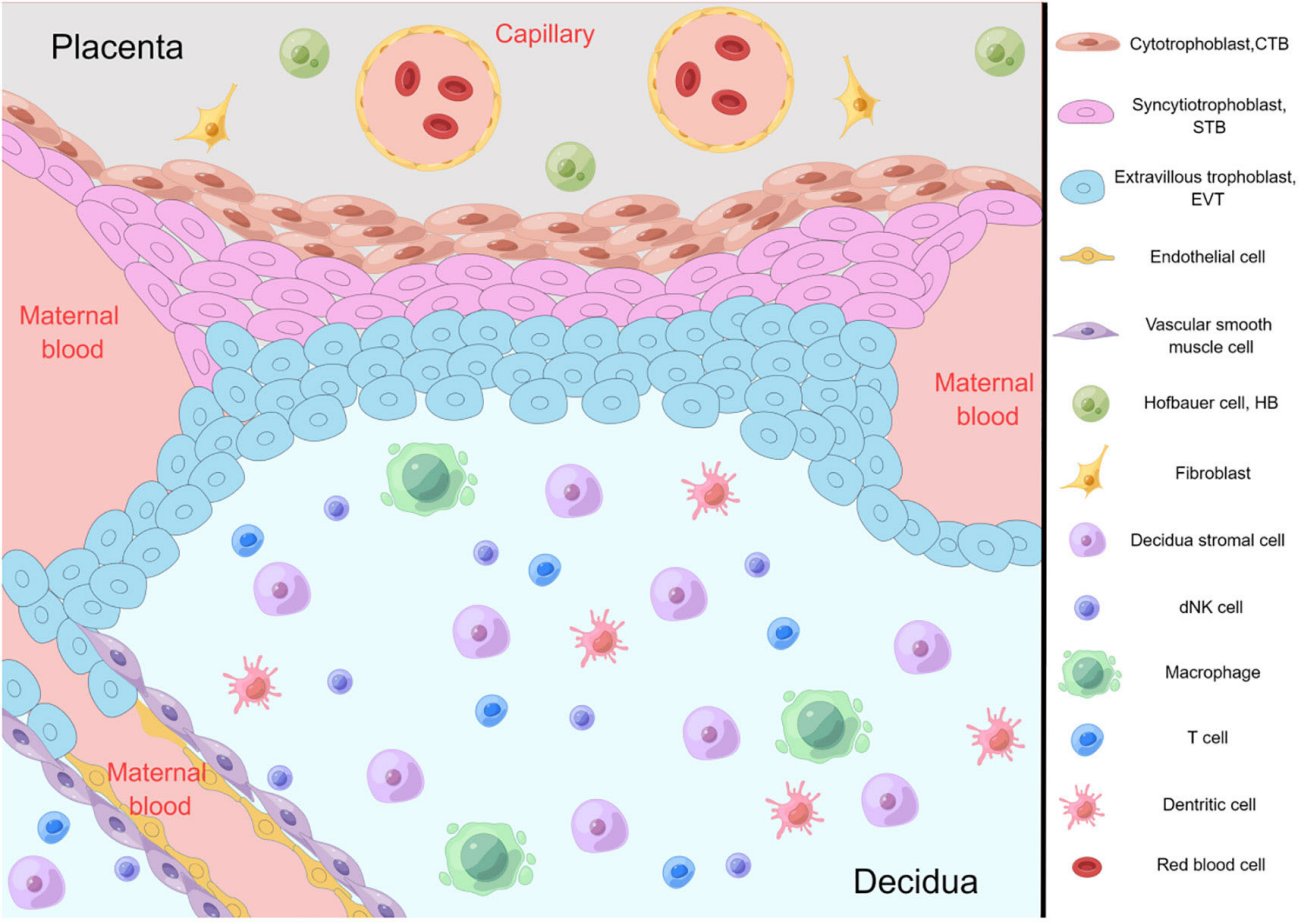
The wisdom of the body working through the cellular components at the maternal-fetal interface.

Exploring gene expression and developmental endocrinology in response to maternal undernutrition stresses the importance of the interplay between maternal health and offspring health outcomes (3). These findings underscore the critical importance of early developmental stages in shaping adult physiological responses. Zhang et al. review the developmental endocrinology of oxidative stress at the maternal-fetal interface (**Figure 1**). They suggest oxidative stress at this site is an important driver of pathology, antioxidant therapy may be the best treatment for “placental diseases”, and an antioxidant lifestyle may help prevent disease. The report thoroughly examines the physiological implications of oxidative stress on the maternal-fetal interface, highlighting the potential ramifications on nutrient transfer, immune regulation, and overall developmental processes. Moreover, it emphasizes the need for continued research endeavors and intervention strategies to mitigate the adverse effects of oxidative stress on this complex interplay, aiming to promote the integrated approach to establishing and maintaining the health of both the expectant mother and the developing fetus.

Thyroid autoimmunity is associated with many maternal and neonatal adverse outcomes (4). In another context of developmental endocrinology in pregnancy, Liu et al. investigate thyroid peroxidase antibodies (TPO-Ab) and their association with first-trimester miscarriage rate/live birth rate in women with unexplained recurrent spontaneous abortion (URSA), which have significant implications for understanding pregnancy outcomes. The findings highlight a higher first-trimester miscarriage rate in TPO-Ab-positive women, particularly in younger subgroups and primary URSA subgroups. While the live birth rate did not exhibit a statistically significant difference between TPO-Ab positive and negative groups, the potential impact of TPO-Ab on pregnancy outcomes, especially in the first trimester, merits further investigation. Acknowledging the study’s limitations, such as its retrospective design, emphasizes the need for larger, prospective randomized studies to confirm the association between TPO-Ab and first-trimester miscarriage rate, particularly in specific subgroups of patients with URSA.

Regarding the role of RORα in developmental endocrinology, Rani reviews the fascinating “staggerer mice” story with one of its first roles materializing during embryogenesis, an intricate molecular-endocrine mediated circadian-like regulatory process. Dysfunctional RORα impairs metabolism, osteogenesis, skeletal and smooth muscles, and immunity, and makes RORα a multi-functional protein during embryogenesis. The text discusses the importance of good nutrition for effective embryonic development and the role of essential nutrients in supporting healthy transcriptional systems. RORα also functions in germ cell organization, another aspect of developmental endocrinology (5).

Adrenal development in embryonic and fetal health expands our understanding of the intricate molecular and physiological processes that shape developmental endocrinology (6). Akkuratova et al. outline a detailed single-cell atlas of chromaffin development, permitting the identification of novel cell populations and establishing nuanced transitions within subpopulations of immature chromaffin cells. The work advances the field of sympatho-adrenal developmental endocrinology. The authors report the discovery of microheterogeneity in developing chromaffin cell populations, the identification of novel markers of adrenergic and noradrenergic populations in developing adrenal glands, and the revelation of new differentiation paths leading to these populations. Additionally, the research emphasized the essential roles of chromaffin cells in fetal survival, the initiation of breathing, and the physiological response to hypoxia. The study’s use of deep single-cell RNA sequencing and trajectory analysis provided valuable insights into the molecular events driving fate choices in Schwann cell precursors and the transient nature of developing chromaffin populations, leading to the identification of previously unknown transient or persisting markers of chromaffin cell subpopulations.

Hypogonadotropic hypogonadism leads to absent, partial, or arrested puberty (7). Zhang et al. provide a comprehensive characterization of Kallmann syndrome and associated genetic variations with the condition. The group provides crucial insights into the genetic and molecular mechanisms underlying this complex disorder, paving the way for precise clinical diagnosis and treatment strategies. The comprehensive study characterized the clinical phenotype and genetic variations in a 14.4-year-old male diagnosed with Kallmann syndrome (KS). Bioinformatics analysis suggested that the IL17RD variant may disrupt fibroblast growth factor signaling by potentially affecting protein phosphorylation and modification. In contrast, the CPEB4 variant appears crucial in affecting olfactory bulb morphogenesis, potentially contributing to the patient’s hyposmia. The study provides valuable insights into the genetic and molecular mechanisms underlying KS. Furthermore, the study broadens the gene expression profile of KS-related pathogenic genes, paving the way for future research in understanding KS pathogenesis. The patient received gonadorelin pump pulse therapy, improving LH, FSH, and T levels. The patient is under ongoing regular follow-up, with follow-up examinations showing noteworthy progress. This study presents significant contributions to the academic understanding of this complex genetic disorder and paves the way for further research in Kallmann syndrome.

Single-cell RNA sequencing is an emerging powerful tool to characterize cell subpopulations, circumventing the shortcomings of traditional cell population sequencing (8). Tirumalasetty et al. provide a comprehensive review of single-cell RNA-sequencing compared to the bulk RNA-seq of rodent and human patients testicular tissues. The team highlights “the cellular heterogeneity, spatial transcriptomics, dynamic gene expression, and cell-to-cell interactions with distinct cell populations within the testes”. The findings have potential implications for future clinical management of male reproductive complications. Liu et al. report a bidirectional cohort study on spermatogenesis and seminal testosterone and offer a potential method to improve the assessment of male infertility and sperm quality. The team concludes measuring testosterone in seminal fluid is more sensitive for judging the presence of local spermatogenesis in nonobstructive azoospermia patients.

These studies collectively contribute to the ongoing dialogue surrounding developmental endocrinology, maternal-fetal health, genetic disorders, and reproductive health. They underscore the importance of continued research efforts to unravel the complex interplay between endocrinological processes, environmental factors, and genetic determinants in shaping developmental outcomes. As we navigate the intricate landscape of developmental endocrinology, this *wisdom of the body perspective* is vital in guiding future research endeavors and clinical interventions to promote maternal and offspring health and well-being.

## Author contributions

LN: Conceptualization, Investigation, Project administration, Supervision, Visualization, Writing – original draft, Writing – review & editing. MC: Writing – review & editing. CR: Writing – review & editing. HK: Writing – review & editing.
